# Atresia of the Aortic Arch in 4-Year-Old Child: A Clinical Case Study

**DOI:** 10.3389/fped.2015.00019

**Published:** 2015-03-20

**Authors:** Vittoria Nigro Stimato, Dominique Didier, Maurice Beghetti, Cécile Tissot

**Affiliations:** ^1^Cardiology Unit, Child and Adolescent Department, Geneva University Hospital, Geneva, Switzerland; ^2^Magnetic Resonance Unit, Department of Radiology, Geneva University Hospital, Geneva, Switzerland

**Keywords:** aortic arch anomalies, atresia aortic arch, magnetic resonance imaging, congenital heart disease, case report

## Abstract

Atresia of the aortic arch is a rare congenital heart defect with a high mortality when associated with other intracardiac defects. Cardiac magnetic resonance (CMR) provides the exact anatomy of the aortic arch and collateral circulation and is useful to diagnose-associated aortic arch anomalies. This report describes the case of a 4-year-old child with atresia of the aortic arch, referred to our institution with the diagnosis of aortic coarctation and bicuspid aortic valve. On clinical exam, the femoral pulses were not palpable and there was a significant differential blood pressure between the upper and lower limbs. The echocardiography showed a severely stenotic bicuspid aortic valve but was limited for the exact description of the aortic arch. CMR showed absence of lumen continuity between the ascending and descending aorta distal to the left subclavian artery, extending over 5 mm, with the presence of a bend in the arch and diverticulum on either side of the zone of discontinuity, suggesting the diagnosis atresia of the aortic arch rather than coarctation or interruption. The patient benefited from a successful surgical commissurotomy of the aortic valve and reconstruction of the aortic arch with a homograft. The post-operative CMR confirmed the good surgical result. This case emphasizes the utility of CMR to provide good anatomical information to establish the exact diagnosis and the operative strategy.

## Introduction

Aortic arch obstructive lesions can have a large spectrum of presentation, going from coarctation through atresia or interruption of the aortic arch. Echocardiography is the exam of choice for assessment of aortic arch anomalies in children, but is sometimes not sufficient in older patients with poor echographic windows. Cardiac magnetic resonance (CMR) provides the exact anatomy of the aortic arch and collateral circulation and is useful to establish the surgical strategy.

## Case Report

We report the case of a 4-year-old boy referred to our institution for surgical treatment of aortic coarctation and bicuspid aortic valve. The patient had no complaint. On clinical exam, the child was in excellent condition. The cardiac exam revealed a harsh 4/6 systolic murmur at the aortic area. The femoral pulses were not palpable, the child was hypertensive on the upper extremities with a significant differential blood pressure between the upper and lower limbs (Table 1).

**Table 1:** **Arterial blood pressure at four limbs in our patient**.

Right upper limb (RUL) 128/82 mmHg	Left upper limb (LUL) 114/80 mmHg
Right lower limb (RLL) 93/71 mmHg	Left lower limb (LLL) 99/61 mmHg

*The differential of blood pressure is maximum 15 mmHg for systolic blood pressure (SBP) and 10 mmHg for diastolic blood pressure (DBP). Normal values at 4 years SBP 102–115 mmHg DBP 62–71 mmHg*.

**Figure 1:**
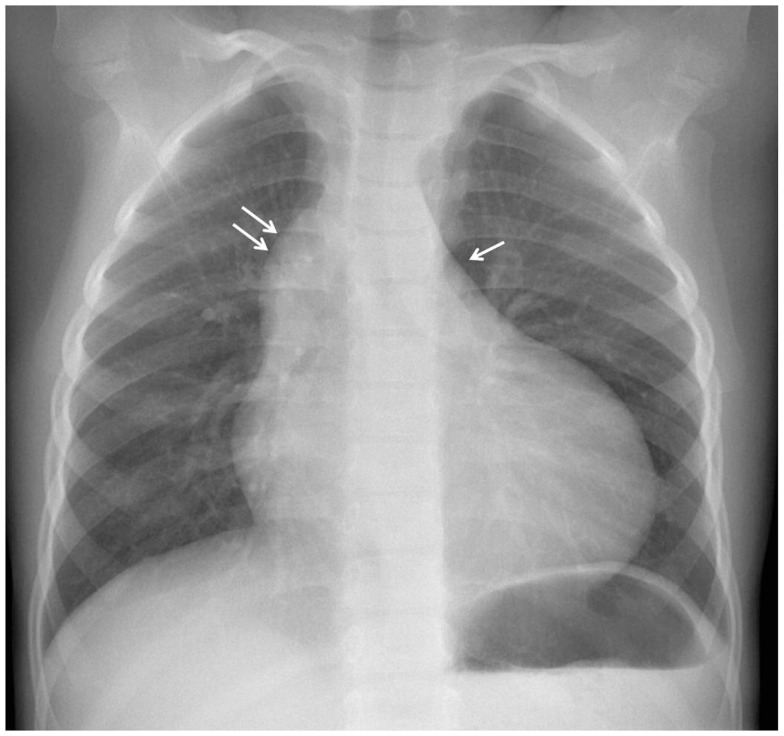
**Chest X-ray with prominent right cardiac border secondary to dilatation of the ascending aorta (double arrow) and absent aortic knob (arrow)**.

**Figure 2:**
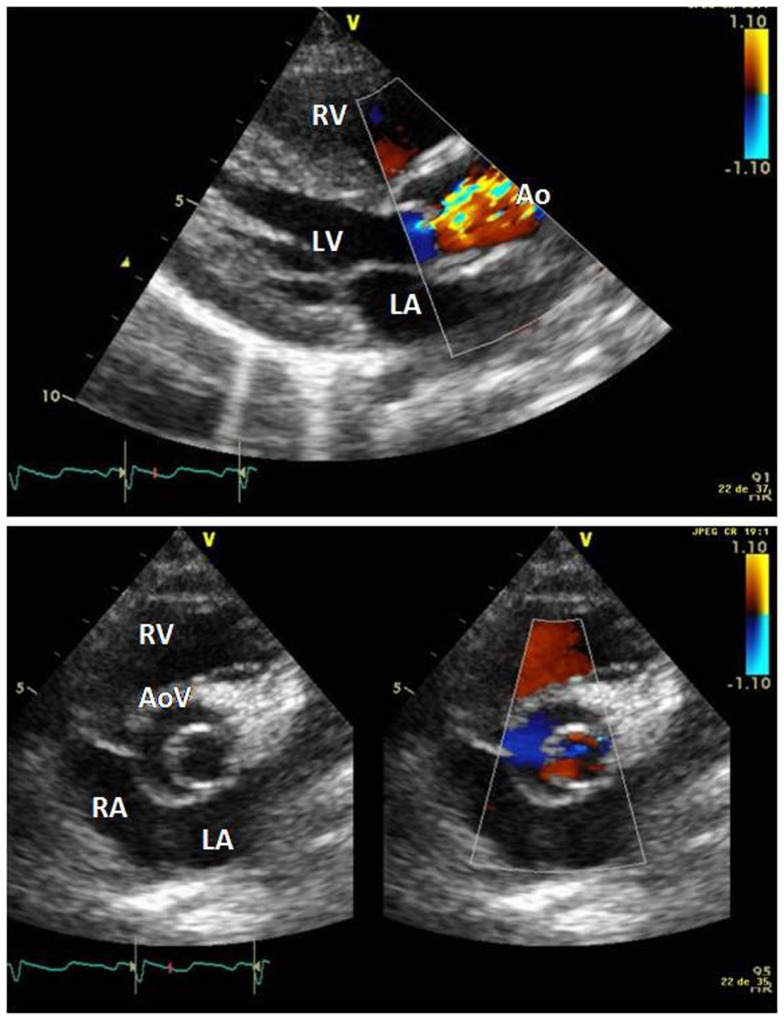
**Long axis parasternal view showing doming of the aortic valve, aliaising of flow secondary to stenosis, and left ventricular hypertrophy**. Short axis view showing bicuspid aortic valve. Abbreviations: AoV, aortic valve; LA, left atrium; LV, left ventricle; RV, right ventricle.

**Figure 3:**
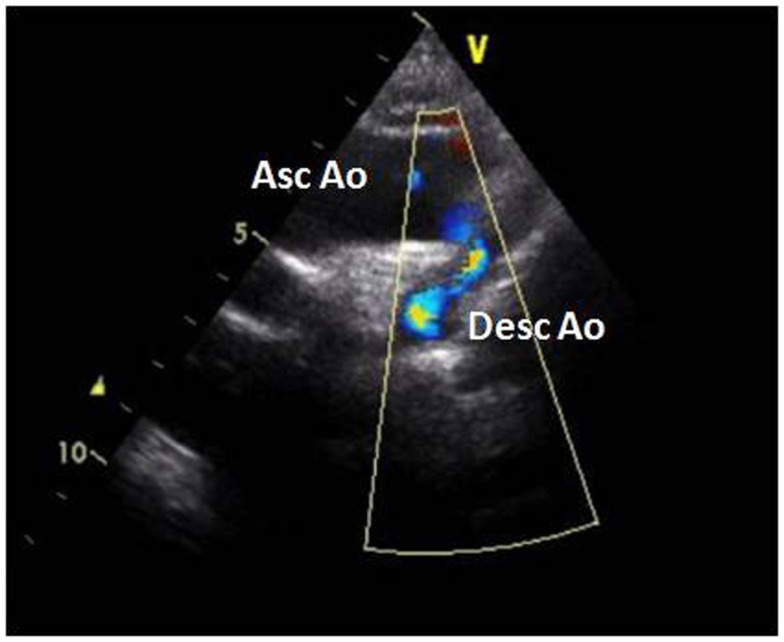
**Suprasternal view of the aortic arch with severe narrowing of the aortic isthmus and aliasing of flow**. Abbreviations: Asc Ao, ascending aorta; Desc Ao, descending aorta.

**Figure 4:**
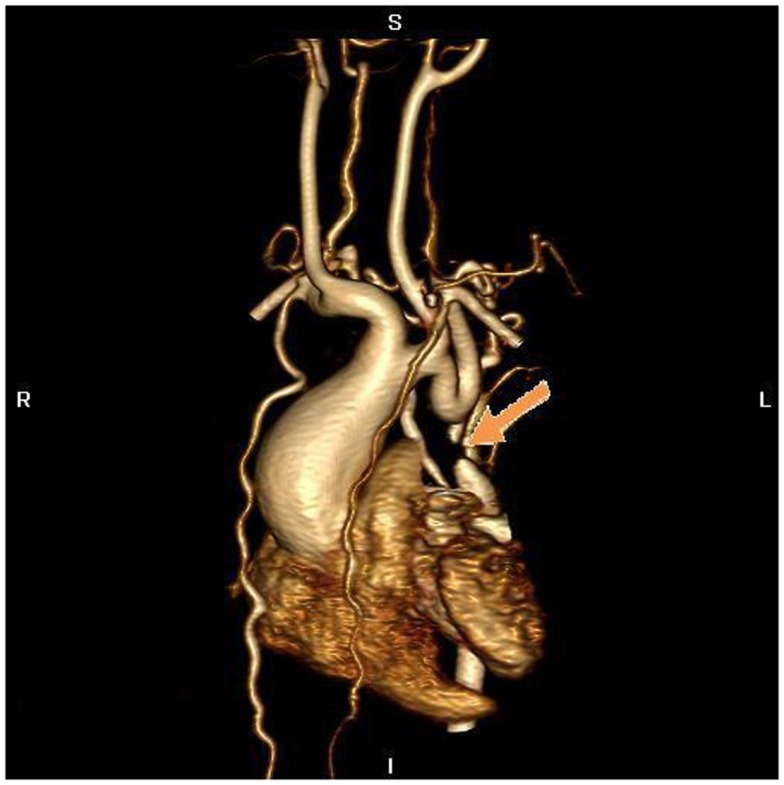
**MR angiography, volume rendering 3D reconstruction**. The arrow indicates the atretic aortic arch, diverticulums on either side of the zone of discontinuity suggests the presence of a fibrous strand at that position, compatible with the diagnosis of atresia. There are numerous collaterals such as both mammary arteries, intercostals arteries, and posterior mediastinal arteries. There was hypoplasia of the horizontal aorta and gothic aspect of the ascending aorta.

**Figure 5:**
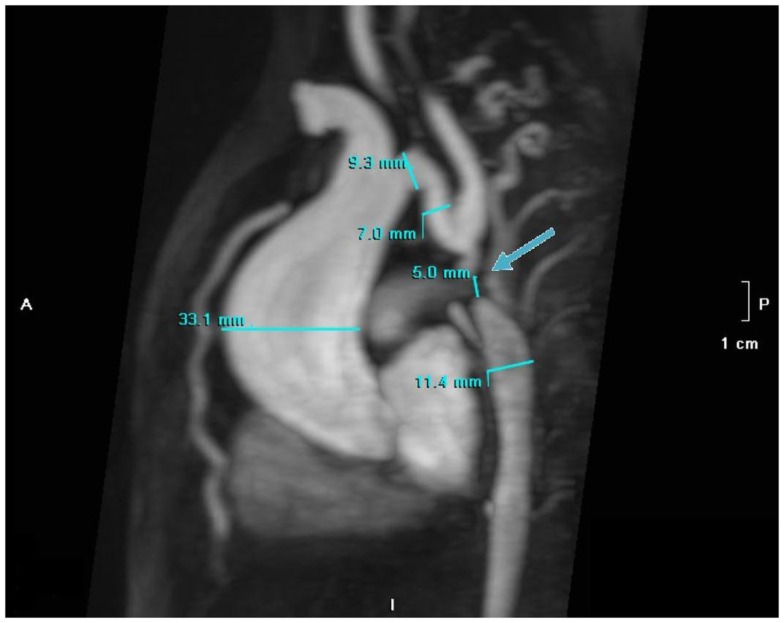
**MR angiography with MIP reconstruction of the atretic aortic arch (5 mm) indicated by the arrow with dilatation (33 mm) of the ascending aorta, secondary to bicuspid aortic valve**.

**Figure 6:**
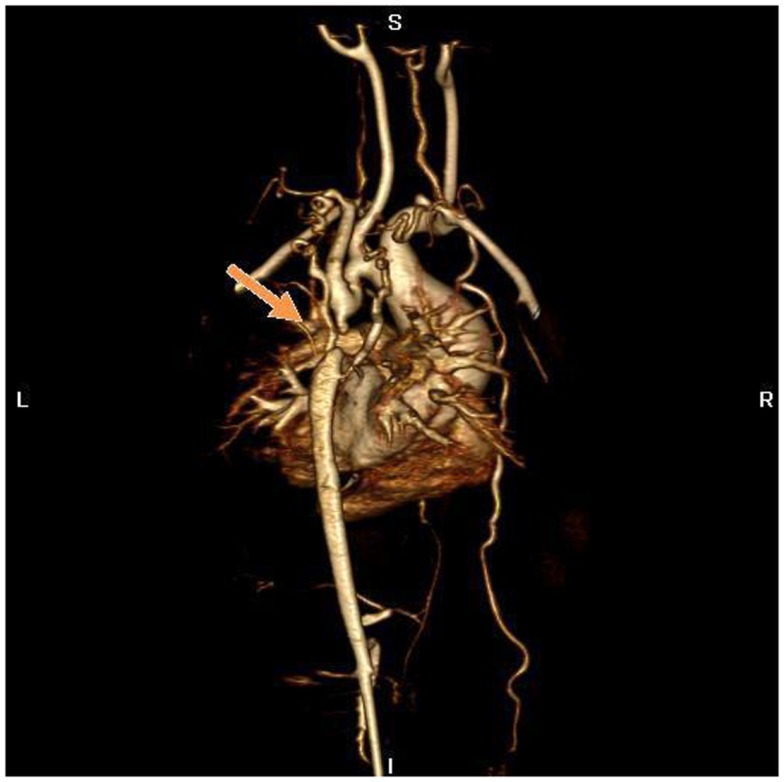
**MR angiography, volume rendering 3D reconstruction**. Posterior view of the aortic arch before surgery.

The electrocardiogram showed sinus rhythm with signs of left auricular hypertrophy, but no ventricular hypertrophy. The chest radiography (Figure 1) showed mild cardiomegaly (ICT 0.64) and an absent aortic knob, compatible with the diagnosis of coarctation. The echocardiography showed a bicuspid aortic valve (Figure 2) with severe stenosis (peak systolic gradient of 60 mmHg and mean of 30 mmHg), left ventricular hypertrophy (*Z*-score +3.3), preserved systolic and diastolic function (mitral E/A ratio 1.6; mitral A deceleration time 0.10 s, E/E′ 22 secondary to increased left heart filling pressure), and tortuous aortic arch with narrowing and acceleration of flow at the aortic isthmus (Figure 3). CMR with angiography showed absence of continuity between the ascending aorta distal to the left subclavian artery (Type 1) and the descending aorta, extending over 5 mm. There was a bend in the arch and diverticulum on either side of the zone of discontinuity, making the diagnosis of atresia rather than interruption of the aortic arch. There was no patent ductus arteriosus (PDA), no associated endofibroelastosis and the descending aorta was fed exclusively by massive systemic collateral circulation (Figures 4–6).

**Figure 7:**
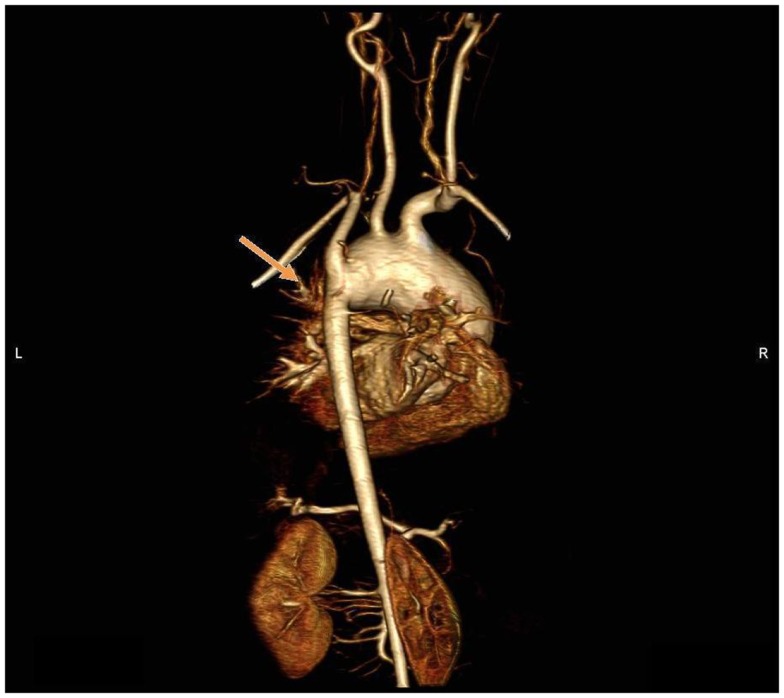
**MR angiography, volume rendering 3D reconstruction**. Posterior view of the aortic arch after surgery.

The patient benefited from successful surgical commissurotomy of the aortic valve and aortic arch reconstruction with interposition of a pulmonary homograft by extended end-to-end anastomosis under cardiopulmonary bypass, with an uneventful post-operative course. The post-operative CMR 1 month after surgery showed good surgical result with patency of the aortic isthmus (Figure 7), and there was no differential in blood pressure between the upper and lower limbs.

## Discussion

Atresia of the aortic arch is a rare congenital anomaly. It is often associated with other cardiac defects, particularly persistent patency of the arterial duct, ventricular septal defect, and sub-aortic or aortic valvar stenosis. Although the distinction between coarctation and interruption is easily made, the difference between interruption and atresia of the aortic arch is not so evident. The two lesions, although producing identical hemodynamic consequences, both produce discontinuity between the transverse and descending components of the aorta. When the aortic arch is interrupted, however, part of its transverse component is completely absent, with varying distance then possible between the ends of the patent components of the arch. When the involved segment is atretic, it remains as an anatomic entity, although being fibrous and lacking any luminal patency. The persisting presence of the fibrous connection between the patent components of the arch also limits the length of the gap between them. In our patient, the presence of a diverticulum on either side of the zone of discontinuity, along with the identification of a fibrous strand by the cardiac surgeon, confirmed the presence of atresia rather than interruption of the aortic arch. The classification into types A, B, and C is used to distinguish the site of both interruption and atresia of the aortic arch. In a large series of patients with interruption, 42% of patients were of type A, with the arch interrupted at the aortic isthmus beyond the origin of the left subclavian artery, 53% of type B, with the interrupted segment between the origin of the left common carotid and left subclavian artery, and 4% of type C, when the interruption was between the innominate and the left common carotid artery (1).

The mortality of interruption or atresia of the aortic arch is high during the first year of life, reaching about 76% without surgical repair (2). Some cases can survive through infancy and late diagnosis has been described in the literature (3–5), almost all late survivors have been shown to have no additional intracardiac anomalies. In this setting, symptoms resemble those of long-standing systemic hypertension and coarctation. Central nervous system symptoms may be seen secondary to vertebral steal, particularly in type B or C. In our patient, the good clinical tolerance by possible slow progression of the aortic valve stenosis in a child in whom obstruction of the aortic arch may have been present soon after birth, in the setting of ductus arteriosus closure. Atresia of the aortic arch may have allowed for collateral vessels formation already during the fetal life, allowing sufficient blood flow supply to the lower part of the body. Some degree of aortic valve stenosis may have been present since fetal life and may have allowed for hypertrophic adaptation of the left ventricle and better hemodynamic tolerance to an associated obstructive lesion of the aortic arch. Similar evolutions have already been described in the literature (6).

Obstructive lesions of the aortic arch are normally diagnosed in the neonatal period by physical examination, but the diagnosis is sometimes missed when the stenosis is mild with a significant number of asymptomatic patients being diagnosed during infancy (7, 8). Echocardiography is a good tool to visualize the aortic arch, particularly in small children, and is most often sufficient to have a good anatomical picture of the arch. CMR particularly 3D gadolinium-enhanced angiography provides the exact anatomy of the aortic arch and collateral circulation (9, 10), and is useful to diagnose-associated aortic arch anomalies like double aortic arch, right-sided aortic arch with mirror image branching and aberrant right subclavian artery (11), essential to establish the preoperative strategy (12). CMR is also important in detecting residual aortic arch hypoplasia, recurrent narrowing and aneurysmal formation in the post-operative period (13) and is recommended by the American Heart Association for every adult patient following aortic root surgery (14). We used CMR in our patient because of poor echographic windows to establish the exact diagnosis, the preoperative strategy, and also to assess the post-operative result.

The impact of bicuspid aortic valve and severe aortic valve stenosis in patients with obstructive lesions of the aortic arch on medium and long-term outcome was recently described by Sugimoto et al. (15), with bicuspid aortic valve representing a risk factor for later regurgitation or stenosis.

## Concluding Remarks

Careful clinical exam is essential in pediatric patients with arterial hypertension and heart murmur to avoid the long-term morbidity associated with undiagnosed obstructive lesions of the aortic arch, the most common diagnosis being coarctation. Echocardiography is essential for the diagnosis but sometimes not sufficient to have a good anatomical description of the anatomy. CMR provides good anatomical information to establish the definitive anatomical diagnosis and the operative strategy.

## Conflict of Interest Statement

The authors declare that the research was conducted in the absence of any commercial or financial relationships that could be construed as a potential conflict of interest.

## References

[1] Van Praagh R Bernhard WF Rosenthal A Parisi LF Fyler DC Interrupted aortic arch: surgical treatment. Am J Cardiol (1971) 27(2):200–11 10.1016/0002-9149(71)90259-1 5100921

[2] Roberts WC Morrow AG Braunwald E Complete interruption of the aortic arch. Circulation (1962) 26:39–59 10.1161/01.CIR.26.1.39 14492799

[3] Pillsbury RC Lower RR Shumway NE Atresia of the aortic arch. Circulation (1964) 30:749–54 10.1161/01.CIR.30.5.749 14226175

[4] Okumura S Niu S Adachi S Ohga K. Adult aortic arch atresia. Jpn J Thorac Cardiovasc Surg (2000) 48(9):599–602. 10.1007/BF03218210 11030136

[5] Milo S Massini C Goor DA Isolated atresia of the aortic arch in a 65-year-old man. Surgical treatment and review of published reports. Br Heart J (1982) 47(3):294–7 10.1136/hrt.47.3.294 7059407 PMC481137

[6] Schumacher G Schreiber R Meisner H Lorenz HP Sebening F Buhlmeyer K. Interrupted aortic arch: natural history and operative results. Pediatr Cardiol (1986) 7(2):89–93. 10.1007/BF02328957 3797292

[7] Vriend JW Lam J Mulder BJ Complete aortic arch obstruction: interruption or aortic coarctation? Int J Cardiovasc Imaging (2004) 20(5):393–6 10.1023/B:CAIM.0000041965.64150.bb 15765862

[8] Ponte M Dias A Dias Ferreira N Fonseca C Mota JC Gama V. Interrupted aortic arch: a misdiagnosed cause of hypertension. Rev Port Cardiol (2014) 33(6):389 e1–5. 10.1016/j.repc.2014.01.014 25001168

[9] Roest AA Helbing WA van der Wall EE de Roos A Postoperative evaluation of congenital heart disease by magnetic resonance imaging. J Magn Reson Imaging (1999) 10(5):656–66 10.1002/(SICI)1522-2586(199911)10:5<656::AID-JMRI8>3.0.CO;2-F 10548773

[10] Ming Z Yumin Z Yuhua L Biao J Aimin S Qian W. Diagnosis of congenital obstructive aortic arch anomalies in Chinese children by contrast-enhanced magnetic resonance angiography. J Cardiovasc Magn Reson (2006) 8(5):747–53. 10.1080/10976640600737425 16891235

[11] Cantinotti M Hegde S Bell A Razavi R. Diagnostic role of magnetic resonance imaging in identifying aortic arch anomalies. Congenit Heart Dis (2008) 3(2):117–23. 10.1111/j.1747-0803.2008.00174.x 18380760

[12] Roche KJ Krinsky G Lee VS Rofsky N Genieser NB. Interrupted aortic arch: diagnosis with gadolinium-enhanced 3D MRA. J Comput Assist Tomogr (1999) 23(2):197–202. 10.1097/00004728-199903000-00006 10096325

[13] Tsai SF Trivedi M Boettner B Daniels CJ. Usefulness of screening cardiovascular magnetic resonance imaging to detect aortic abnormalities after repair of coarctation of the aorta. Am J Cardiol (2011) 107(2):297–301. 10.1016/j.amjcard.2010.09.016 21211607

[14] Warnes CA Williams RG Bashore TM Child JS Connolly HM Dearani JA ACC/AHA 2008 guidelines for the management of adults with congenital heart disease: a report of the American College of Cardiology/American Heart Association Task Force on Practice Guidelines (Writing Committee to Develop Guidelines on the Management of Adults With Congenital Heart Disease). Developed in Collaboration With the American Society of Echocardiography, Heart Rhythm Society, International Society for Adult Congenital Heart Disease, Society for Cardiovascular Angiography and Interventions, and Society of Thoracic Surgeons. J Am Coll Cardiol (2008) 52(23):e143–263. 10.1016/j.jacc.2008.10.001 19038677

[15] Sugimoto A Ota N Miyakoshi C Murata M Ide Y Tachi M Mid- to long-term aortic valve-related outcomes after conventional repair for patients with interrupted aortic arch or coarctation of the aorta, combined with ventricular septal defect: the impact of bicuspid aortic valve. Eur J Cardiothorac Surg (2014) 46(6):952–60. 10.1093/ejcts/ezu078 24616392

